# Low E2F1 transcript levels are a strong determinant of favorable breast cancer outcome

**DOI:** 10.1186/bcr1681

**Published:** 2007-05-29

**Authors:** Vincent Vuaroqueaux, Patrick Urban, Martin Labuhn, Mauro Delorenzi, Pratyaksha Wirapati, Christopher C Benz, Renata Flury, Holger Dieterich, Frédérique Spyratos, Urs Eppenberger, Serenella Eppenberger-Castori

**Affiliations:** 1 Stiftung Tumorbank Basel, Lörracherstrasse 50, 4125 Riehen, Switzerland; 2 OncoScore AG, Lörracherstrasse 50, 4125 Riehen, Switzerland; 3 Swiss Institute for Experimental Cancer Research and Swiss Institute of Bioinformatics, Boveresses 155, Office D208, CH-1006 Epalinges, Switzerland; 4 Chemin des Boveresses 155, CH-1066 Epalinges, Switzerland; 5 Room 2021, Genopode, UNIL Sorge, CH-1015 Lausanne, Switzerland; 6 Therapeutics Buck Institute for Age Research 8001 Redwood Blvd., Novato, CA 94945, USA; 7 Fachärztin FMH für Pathologie, Chefärztin Pathologie, Brauerstrasse 15,8401 Winterthur, Switzerland; 8 Women's Hospital and Breast Cancer Center, Therese-Herzog-Weg 2, 79618 Rheinfelden, Germany; 9 Laboratoire d'Oncogénétique/INSERM U735, Centre René Huguenin, 35 rue Dailly, 92210 St-Cloud, France

## Abstract

**Introduction:**

We investigated whether mRNA levels of E2F1, a key transcription factor involved in proliferation, differentiation and apoptosis, could be used as a surrogate marker for the determination of breast cancer outcome.

**Methods:**

E2F1 and other proliferation markers were measured by quantitative RT-PCR in 317 primary breast cancer patients from the Stiftung Tumorbank Basel. Correlations to one another as well as to the estrogen receptor and ERBB2 status and clinical outcome were investigated. Results were validated and further compared with expression-based prognostic profiles using The Netherlands Cancer Institute microarray data set reported by Fan and colleagues.

**Results:**

E2F1 mRNA expression levels correlated strongly with the expression of other proliferation markers, and low values were mainly found in estrogen receptor-positive and ERBB2-negative phenotypes. Patients with low E2F1-expressing tumors were associated with favorable outcome (hazard ratio = 4.3 (95% confidence interval = 1.8–9.9), *P *= 0.001). These results were consistent in univariate and multivariate Cox analyses, and were successfully validated in The Netherlands Cancer Institute data set. Furthermore, E2F1 expression levels correlated well with the 70-gene signature displaying the ability of selecting a common subset of patients at good prognosis. Breast cancer patients' outcome was comparably predictable by E2F1 levels, by the 70-gene signature, by the intrinsic subtype gene classification, by the wound response signature and by the recurrence score.

**Conclusion:**

Assessment of E2F1 at the mRNA level in primary breast cancer is a strong determinant of breast cancer patient outcome. E2F1 expression identified patients at low risk of metastasis irrespective of the estrogen receptor and ERBB2 status, and demonstrated similar prognostic performance to different gene expression-based predictors.

## Introduction

A variety of genes involved in breast cancer biology have been studied and proposed as prognostic or predictive biomarkers, but only a few of them, such as hormone receptors and ERBB2, are used today to classify breast cancer patients and to make treatment decisions in the clinical routine [1,2]. The introduction of microarray analysis recently lead to a better characterization of breast cancer on a molecular level, underlining its biological heterogeneity and revealing that breast tumors can be grouped into different subtypes with distinct gene expression profiles and prognosis [3]. Some of these subtypes confirmed the relevance of established differences between phenotypes such as the estrogen receptor (ER) and ERBB2 status, but also identified novel breast cancer subtypes or prognostic signatures of potential clinical value [3-7]. Although little overlap was observed between these gene signatures at the level of individual genes, recent data indicate that the underlying biological processes and pathways might be common [8-10].

In terms of tumor biology, proliferation has been recognized as a distinct hallmark of cancer and as an important determinant of cancer outcome [11-13]. Increased tumor cell proliferation is accompanied by cell matrix remodeling and neo-angiogenesis, which together form the basis for an aggressive tumor phenotype [14,15]. This observation was further underlined by recent reports showing that several genes involved in gene signatures discriminating clinically relevant breast cancer subtypes were related to proliferation [3,4,9,16,17].

In the context of breast cancer molecular screening, we recently investigated by quantitative RT-PCR the expression of 60 tumor-related genes in various subsets of breast cancers from the Stiftung Tumorbank Basel (STB) [18,19]. This gene set also comprised several genes involved in proliferation such as thymidilate synthase (TYMS), thymidine kinase 1 (TK1), topoisomerase 2-alpha (TOP2A), survivin (BIRC5) and the transcription factor E2F1. Since these genes strongly correlated to one another and since the assessment of a single gene able to accurately predict breast cancer patients' outcome would represent major advantages for standard clinical use, we focused our efforts on the evaluation of E2F1 transcript levels as surrogate marker for proliferation. This transcription factor is well known for being involved in the cyclin/cyclin-dependent kinase/retinoblastoma pathway and for controlling the expression of more than 1,000 genes involved in cell proliferation, differentiation and apoptosis [20-23]. In a set of 317 primary breast cancers patients with known clinical outcome (STB data set), we evaluated E2F1 mRNA expression levels with respect to other proliferation markers, ER and ERBB2 status and clinical outcome. All results obtained in our collective were subsequently validated in The Netherlands Cancer Institute (NKI) microarray data set comprising 295 breast cancer patients. Moreover, the prognostic value of E2F1 was compared with the 70-gene prognostic signature, and with other gene expression-based predictors such as the intrinsic subtypes, the wound response signature and the recurrence score available as reported by Fan and colleagues using the same NKI data set [8].

## Methods

### Study populations

Patients and methods have been described previously [18]. The 317 primary breast cancer tissue samples were obtained from the STB, Switzerland and were analyzed by quantitative RT-PCR (STB data set). The previously published microarray breast cancer data set reported by Van de Vijver and colleagues (NKI data set) [5] was used for validation and comparative analysis as reported by Fan and colleagues [8]. Major differences between the two study populations included the patient age, nodal status, adjuvant therapy and methodology (quantitative RT-PCR versus Agilent microarray). Detailed patient and tumor characteristics are summarized in Table 1.

**Table 1:** Patient and tumor characteristics

Characteristic	Stiftung Tumorbank Basel data set	The Netherlands Cancer Institute data set
Method	Quantitative RT-PCR	Agilent Microarray
*n*	317	295
Age		
Mean/median (years)	60/59	44/44
≤ 40 years	20 (6%)	75 (25%)
41–55 years	110 (35%)	220 (75%)
≥ 56 years	187 (59%)	0 (0%)
pT stage		
pT1	100 (32%)	155 (53%)
pT2	183 (58%)	140 (47%)
pT3/4	33 (10%)	0
pN status		
Negative	161 (54%)	151 (51%)
Positive	136 (46%)	144 (49%)
Unknown	20	0
Histological grade		
1 (good)	28 (9%)	75 (25%)
2 (intermediate)	137 (46%)	101 (34%)
3 (poor)	133 (45%)	119 (41%)
Unknown	19	0
Estrogen receptor status^a^		
Positive	231 (73%)	226 (77%)
Negative	86 (27%)	69 (23%)
ErbB2 status		
Positive	70 (22%)	52 (18%)
Negative	247 (78%)	243 (82%)
Adjuvant therapy		
None	60 (20%)	165 (56%)
Hormone	135 (44%)	20 (7%)
Chemotherapy	72 (24%)	90 (30%)
Combination	38 (12%)	20 (7%)
Total	245 (80%)	130 (44%)
Unknown	12	0
Follow-up		
Events (metastases)	57 (18%)	101 (34%)
Mean/median metastasis-free survival (years)	3.7/3.6	7.3/6.8

Data presented as *n *(%) unless stated otherwise. ^a^Estrogen receptor positive, ≥ 20 fmol/mg protein (enzyme immunoassay) for the Stiftung Tumorbank Basel data set; for The Netherlands Cancer Institute study, see [5,6]

### Quantitative real-time PCR analysis

Gene expression measurements by quantitative RT-PCR were performed as reported previously [24]. Total RNA was extracted using the RNAeasy Mini Kit (Qiagen, Hilden, Germany) and was quality-checked on a Bioanalyzer 2100 (Agilent Technologies, Palo Alto, CA, USA). High-quality RNA samples were reverse-transcribed and PCR was carried out in 40 cycles on a ABI Prism 7000 using 2× SYBR Green I Master Mix (Applied Biosystems, Forster City, CA, USA). Relative gene expression quantities (Δ[Ct] values) were obtained by normalization against ribosomal 18S RNA.

### Statistical analysis

For the STB study the ER status was defined based on the mRNA level as reported previously [24], and for the NKI data set the status was defined as provided by the authors [5,8]. The ERBB2 status was determined in both the STB and NKI data sets using mRNA expression levels for all study populations as previously described by Urban and colleagues [18].

The prognostic value of biomarkers was assessed by univariate and multivariate Cox analysis against metastasis-free survival (MFS), and in different patient subgroups according to the ER and ERBB2 status. The association of E2F1 with MFS in particular was assessed by univariate Cox analysis for various cutoff values (data not shown). For all subsequent analysis, the 30th percentile was used as the cutoff point for E2F1. Survival probabilities for MFS were calculated according to the Kaplan–Meier method, and group differences were assessed by the logrank test. Multivariate *P *values were based on Wald statistics. Statistical analysis was performed with 'R' statistical software version 2.0.1 using the 'survival' package [25].

## Results

### E2F1 correlated with other proliferation markers and clinical outcome

A strong and significant correlation was found between the five proliferation markers analyzed in the STB data set (Table 2). Univariate Cox regression analysis demonstrated a significant association of E2F1 as well as TYMS, TK1, TOP2A and BIRC5 expression levels with distant MFS (Table 2). Similar results were observed in the NKI data set (data not shown). In the NKI data set we also investigated Ki67. The RNA expression levels of this proliferation marker were positively correlated with E2F1 (correlation coefficient = 0.46) and were borderline significant (*P *= 0.02) in univariate Cox regression analysis.

**Table 2:** Correlation among different proliferation markers in the Stiftung Tumorbank Basel data set and association with survival

	Correlation^a^				Univariate Cox regression^b ^(*P *value)
		
	E2F1	BIRC5	TOP2A	TK1	
E2F1	-				<0.001
BIRC5	0.84				0.001
TOP2A	0.78	0.76			<0.001
TK1	0.79	0.88	0.67		0.018
TYMS	0.81	0.80	0.71	0.77	0.005

^a^Pearson correlation coefficient, all *P *< 0.05. ^b^Metastasis-free survival.

### Distinct E2F1 expression patterns according to ER and ERBB2 status determined the clinical outcome

**Figure 1:**
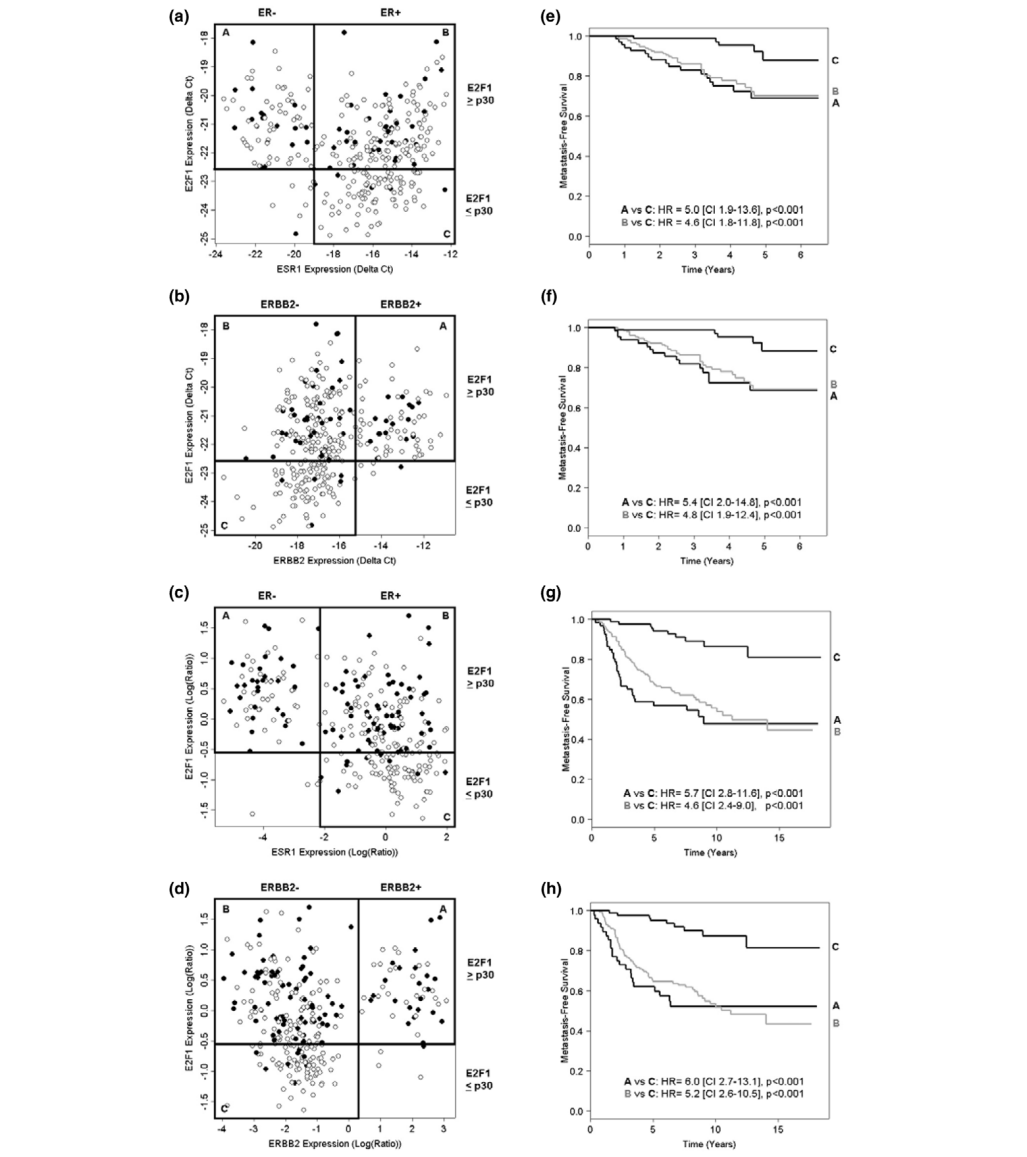
**Estrogen receptor and ERBB2 versus E2F1 expression levels**. Scatter plots of estrogen receptor (ER) ESR1 and ERBB2 versus E2F1 expression levels in **(a), (b) **the Stiftung Tumorbank Basel data (STB) set and **(c), (d) **The Netherlands Cancer Institute (NKI) data set. Open circles, no metastasis; filled circles, metastasis. Vertical lines, cutoff values for the estrogen receptor (ER) and ERBB2 status, respectively; horizontal lines, 30th percentile for E2F1. Combined Kaplan–Meier analysis (metastasis-free survival) using the ER or ERBB2 status and E2F1 (30th percentile) in **(e), (f) **the STB data set and **(g), (h) **the NKI data set. Labels of the survival curves correspond to the groups as indicated on the respective scatter plot. CI, 95% confidence interval; HR, hazard ratio.

Scatter plots of E2F1 versus ER and ERBB2 expression levels in the STB data set (Figure 1a,b) revealed that ER-negative and ERBB2-positive breast tumors typically expressed high levels of E2F1, whereas in contrast low E2F1 levels (below the 30th percentile of its distribution in this collective) were detected almost exclusively in ER-positive and ERBB2-negative breast tumors. The same pattern was observed in the NKI data set (Figure 1c,d). Similar scatter plots were obtained analyzing the other proliferation markers (data not shown).

Cox univariate survival analysis performed in subsets of patients according to their ER and ERBB2 status showed that E2F1 correlated with MFS in ER-positive and ERBB2-negative tumors, but not in ER-negative and ERBB2-positive tumors (data not shown). Combined Kaplan–Meier analysis using E2F1 and the ER or ERBB2 status revealed that patients whose tumors expressed low E2F1 levels, a situation found mainly in ER-positive and ERBB2-negative phenotypes, were associated with favorable outcome, whereas patients with tumors expressing high E2F1 levels revealed a poor outcome independent of the ER and ERBB2 status (Figure 1e-h).

### E2F1 correlated well with the 70-gene signature

**Figure 2:**
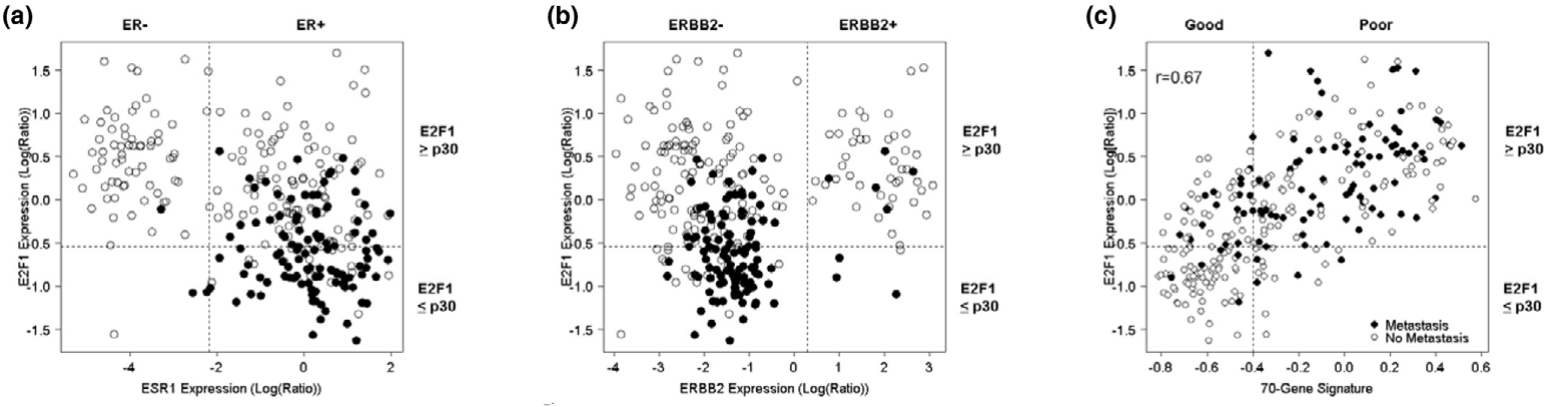
**Comparison of E2F1 and the 70-gene signature in The Netherlands Cancer Institute data set**. **(a), (b) **Scatter plots of estrogen receptor (ER = ESR1) and ERBB2 versus E2F1 expression levels. Open circles, poor-prognosis group as defined by [5]; filled circles, good-prognosis group [5]. **(c) **Correlation between the 70-gene prognostic signature and E2F1. Open circles, no metastasis; filled circles, metastasis.

The majority of the patients in the NKI data set assigned to the good-prognosis group by the 70-gene signature expressed low E2F1 levels and were found to be ER-positive or ERBB2-negative (Figure 2a,b). In addition, there was a strong correlation (*r *= 0.67) between E2F1 and the 70-gene signature (Figure 2c). In particular, 77% (69 out of 90) of patients with low E2F1-expressing tumors overlapped with patients assigned to the good-prognosis group by the 70-gene signature and were indeed found to be at the lowest risk of metastatic events. Patients with low E2F1 and a poor-prognosis signature or patients with high E2F1 and a good-prognosis signature had a comparable incidence of metastases (Table 3).

**Table 3:** Concordance of E2F1 with the 70-gene signature in The Netherlands Cancer Institute data set

Proliferation status (E2F1)	NKI 70-gene signature prognosis		
	
	Good	Poor	Total
E2F1 ≤ p30	4/69 (5.7%)	6/21 (28.6%)	10/90 (11.1%)
E2F1 > p30	12/46 (26.1%)	79/159 (49.6%)	91/205 (44.4%)
Total	16/115 (13.9%)	85/180 (47.2%)	101/295 (34.2%)

The percentage indicates the number of metastatic events over the number of cases in each group.

### E2F1 stratification showed similar prognostic value as the 70-gene and other gene-based predictors

**Figure 3:**
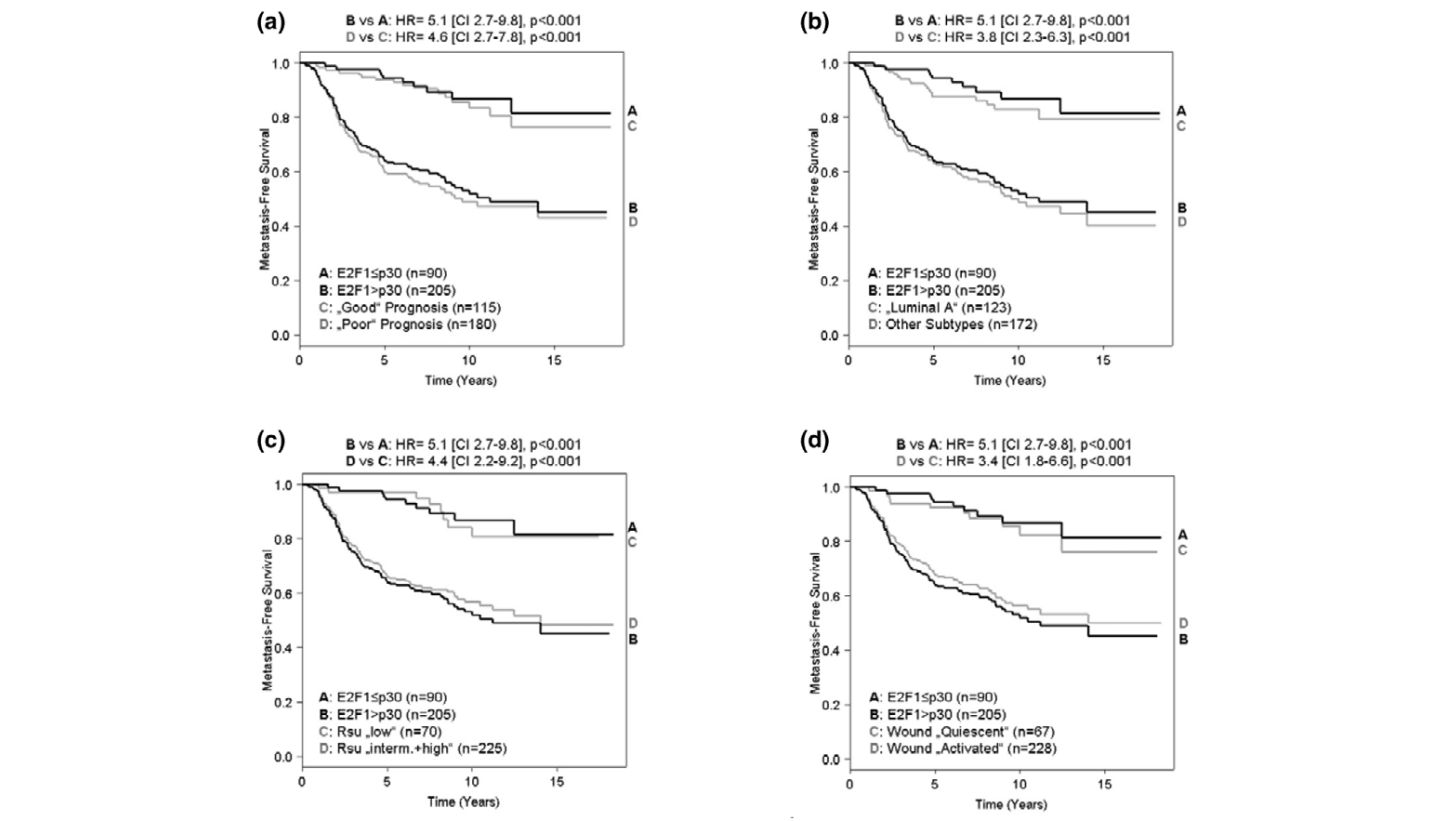
**Kaplan–Meier analysis of metastasis-free survival**. Kaplan–Meier analysis (metastasis-free survival) using **(a) **E2F1 expression (30th percentile) and the 70-gene signature, **(B) **intrinsic subtypes, **(c) **the recurrence score (Rsu), and **(b) **the wound response signature. CI, 95% confidence interval; HR, hazard ratio.

Kaplan–Meier analysis displayed the similar prognostic value of E2F1 and the 70-gene signature (hazard ratio = 5.1 (95% confidence interval = 2.7–9.8) and hazard ratio = 4.6 (95% confidence interval = 2.7–7.8), respectively; Figure 3a). We obtained similar results (Figure 3b–d) when E2F1 levels were compared with the breast cancer intrinsic subtypes [3], with the recurrence score [17] and with the wound response signature [7], all of these gene expression-based predictors being reported by Fan and colleagues in the NKI data set [8].

### E2F1 was a strong and independent survival factor in multivariate analysis

Multivariate survival analysis including E2F1, nodal status, grade, tumor size, age, ER and ERBB2 status, and treatments revealed that only E2F1 and nodal status retained independent prognostic value in the STB data set (Table 4), and that E2F1, nodal status, tumor size, age and chemotherapy were significant in the NKI data set (Table 5). We performed a second multivariate Cox model including additionally the 70-gene signature in the NKI data set (Table 5), reconfirming that E2F1 and the 70-gene signature were significant and additive predictive survival factors together with the nodal status, tumor size and chemotherapy.

**Table 4:** Univariate and multivariate Cox analyses in the Stiftung Tumorbank Basel data set (*n *= 317)

Factor	Univariate metastasis-free survival		Multivariate metastasis-free survival	
	
	Hazard ratio (95% confidence interval)	*P *value	Hazard ratio (95% confidence interval)	*P *value
E2F1 (>p30 versus ≤ p30)	4.27 (1.83–9.96)	0.001	2.95 (1.10–7.93)	0.032
Grade (3 versus 1 + 2)	2.03 (1.17–3.51)	0.011	1.56 (0.77–3.14)	0.213
Estrogen receptor status (positive versus negative)	0.64 (0.37–1.12)	0.120	1.21 (0.56–2.61)	0.625
ERBB2 status (positive versus negative)	1.69 (0.98–2.92)	0.058	1.41 (0.70–2.81)	0.335
pN (>3 nodes versus ≤ 3 nodes)	3.14 (1.76–5.61)	<0.001	2.36 (1.14–4.88)	0.021
Size (>2 cm versus ≤ 2 cm)	1.95 (1.05–3.62)	0.036	1.19 (0.58–2.43)	0.641
Age (≤ 40 years versus >40 years)	0.30 (0.15–0.59)	0.001	0.59 (0.24–1.43)	0.246
Chemotherapy	2.65 (1.54–4.55)	<0.001	1.27 (0.45–3.59)	0.654
Hormone therapy	0.50 (0.28–0.88)	0.017	0.76 (0.27–2.17)	0.605
70-gene signature (poor versus good prognosis)	Not available	Not available	Not available	Not available

**Table 5:** Univariate and multivariate Cox analyses in The Netherlands Cancer Institute data set (*n *= 295)

Factor	Univariate metastasis-free survival		Multivariate metastasis-free survival^a^			
	
	Hazard ratio (95% confidence interval)	*P *value	With 70-gene signature		Without 70-gene signature	
			
			Hazard ratio (95% confidence interval)	*P *value	Hazard ratio (95% confidence interval)	*P *value
E2F1 (>p30 versus ≤ p30)	5.09 (2.65–9.78)	<0.001	3.76 (1.90–7.45)	<0.001	2.47 (1.20–5.10)	0.014
Grade (3 versus 1 + 2)	2.38 (1.60–3.52)	<0.001	1.28 (0.82–2.00)	0.277	1.02 (0.65–1.60)	0.931
Estrogen receptor status (positive versus negative)	0.54 (0.36–0.83)	0.005	0.99 (0.61–1.59)	0.956	1.11 (0.70–1.78)	0.658
ERBB2 status (positive versus negative)	1.61 (1.01–2.57)	0.045	1.35 (0.82–2.21)	0.234	1.28 (0.78–2.09)	0.330
pN (>3 nodes versus ≤ 3 nodes)	2.20 (1.37–3.53)	0.001	2.35(1.32–4.21)	0.004	2.69 (1.47–4.91)	0.001
Size (>2 cm versus ≤ 2 cm)	2.08 (1.39–3.10)	<0.001	1.70 (1.12–2.58)	0.013	1.73 (1.14–2.62)	0.010
Age (≤ 40 years versus >40 years)	0.50 (0.33–0.75)	0.001	0.59 (0.38–0.89)	0.013	0.67 (0.43–1.02)	0.063
Chemotherapy	0.79 (0.52–1.19)	0.254	0.61 (0.38–1.00)	0.051	0.55 (0.33–0.91)	0.020
Hormone therapy	0.58 (0.28–1.19)	0.139	0.60 (0.28–1.27)	0.181	0.58 (0.27–1.23)	0.157
70-gene signature (poor versus good prognosis)	4.55 (2.67–7.77)	<0.001	Not included	Not included	2.78 (1.49–5.21)	0.001

^a^Multivariate metastasis-free survival calculated once with and once without the 70-gene signature.

## Discussion

In the present study we demonstrated that the assessment of E2F1 mRNA as a surrogate proliferation marker is a strong determinant of breast cancer outcome, particularly suitable for identifying patients at very low risk of metastasis, comparable with gene expression-based signatures such as the 70-gene signature. The prognostic component of the ER and ERBB2 status as well as different gene signatures were found to be strongly related to tumor proliferation. In fact, a large subset of patients classified with very favorable outcome shared a common molecular tumor phenotype characterized by ER-positive and/or ERBB2-negative status and low proliferation (low levels of E2F1 as well as *BIRC5*,*TYMS*,*TOP2A *and *TK1*). Moreover, the results obtained in our data set analyzed by quantitative RT-PCR were successfully validated in an independent breast cancer data set using microarray technology.

Sotiriou and colleagues developed a gene expression grade index able to reclassify breast cancer patients with tumor histological grade 2 into groups with high risk of recurrence versus low risk [9]. The gene expression grade index was developed on the basis of the analysis of five breast cancer microarray data sets including more than 600 tumors, from which the authors extracted a list of 242 genes associated with tumor grade and predicting patient outcome. Most of these genes were related to proliferation and cell survival, such as E2F1 and MKI67, BIRC5, TOP2A and STK6, all being highly correlated and providing similar prognostic information. In our study, we demonstrated that the detection of a single gene is sufficient to select tumors at low proliferation. A single gene assessment requires high RNA quality from fresh (frozen) tissue, however, and might be insufficient in cases of more heterogeneous RNA quality (for example, RNA from paraffin-embedded tissues).

Breast cancer has been successfully classified using microarrays into clinically relevant subgroups based on variations in gene expression patterns. Sorlie and colleagues showed that ER-negative tumors grouped into basal-like and ERBB2 subtypes, both with poor prognosis [3]. In contrast, ER-positive breast cancers could be classified into luminal A and luminal B subtypes with significantly distinct prognosis: luminal A tumors displayed favorable outcome, whereas survival of patients with luminal B tumors was poor and comparable with those of the ER-negative ERBB2 and basal subtypes [3]. Our classification in the NKI data set revealed that 81% of the tumors expressing low E2F1 levels (below this study's cutoff point) corresponded with luminal A subtype as defined by Fan and colleagues [8], and subsequently had similar prognostic value (Figure 3b).

Van de Vijver and colleagues used a 70-gene prognostic signature to discriminate patients with good prognosis and poor prognosis [5], which according to our analysis strongly correlated with E2F1 expression levels. As shown in Figure 2, patients defined as of good prognosis by the 70-gene signature had tumors expressing low E2F1 levels and were mainly ER-positive. Despite all observed correlations, multivariate Cox analysis of the NKI data set showed that E2F1 levels and the 70-gene prognostic signature retained additive significance when both covariates were included (Table 5). This is probably due to the fact that both markers classified, in addition to the overlapping patients at very low risk, patients at similar but higher risk who would not have been selected by either classifier alone (Table 3). Furthermore, we found that almost all ERBB2-positive and ER-negative tumors expressed high levels of E2F1 and were classified as of poor prognosis according to the 70-gene signature – suggesting an explanation of why Espinosa and colleagues were unsuccessful in improving the accuracy of the 70-gene signature by incorporating additional genes such as ERBB2 [26].

Fan and colleagues [8] recently demonstrated that the different gene-expression-based predictors including the 70 gene-signature, the intrinsic subtypes, the wound signature and the recurrence score were highly concordant to evaluate breast cancer outcome. Our analysis revealed that low proliferation as quantified by low levels of E2F1 represented a common determinant of patients with good prognosis (Figures 2 and 3). It has to be noted that the prognostic value of E2F1 was independent of the nodal status. Indeed, 40% of the STB tumors and 50% of the NKI tumors with low E2F1 expression levels belonged to nodal-positive patients at very low risk of metastases, reconfirming the impact of proliferation recently reported in a study evaluating breast cancer patients with 10 and more positive lymph nodes [27,28].

The STB and NKI data sets differed in adjuvant treatment modalities; in general, patients of the STB collective were older and consequently received more hormone therapy but less chemotherapy as compared with patients of the NKI collective. In this context, it has to be emphasized that treatment regiments were chosen independent of the E2F1 status (Additional file 1) and that E2F1 levels retained predictive survival significance in patients with and without different adjuvant treatments (Additional file 2). Multivariate analyses, however, revealed different treatment impacts in the two data sets (Tables 4 and 5). In the STB collective, chemotherapy was particularly significant in univariate Cox analysis but was nonsignificant in multivariate Cox models, suggesting that information about the higher risk cases receiving chemotherapy is already included in the combination of the other covariates. Since E2F1 is co-expressed or regulates genes such as TYMS, TK1 and TOP2A, which were mechanistically linked with response to 5-fluorouracil and anthracycline-based therapy [16,29-32], however, our results with respect to specific chemotherapy response should be further investigated.

## Conclusion

Since accurate monitoring of proliferation assessing the mRNA E2F1 levels together with the determination of the ER and ERBB2 status can be performed easily by quantitative RT-PCR even in small amounts of tissue such as core biopsies [19], we encourage the inclusion of such analyses in protocols of ongoing clinical and translational research investigations, including predictive studies with respect to specific chemotherapies.

## Abbreviations

ER = estrogen receptor; MFS = metastasis-free survival; NKI = The Netherlands Cancer Institute; PCR = polymerase chain reaction; RT = reverse transcriptase; STB = Stiftung Tumorbank Basel.

## Competing interests

The authors declare that they have no competing interests.

## Authors' contributions

VV, ML and SE-C designed the study. VV and ML contributed to the selection of the genes, selected primers, and supervised the RNA extraction and quantitative RT-PCR. VV and PU performed statistical analysis under the supervision of MD and PW. VV, PU, MD, PW and SE-C contributed to data interpretation. RF performed the pathological analysis of several samples and asserved the surgical samples for molecular analysis. CCB, HD, RF, FS and UE participated in designing the study and writing the manuscript. VV, PU and SE drafted the manuscript. All authors read and approved the final manuscript.

## Supplementary Material

Additional File 1 A Word file containing a table presenting the treatment distribution according to the E2F1 status in both data sets. Click here for file

Additional File 2 A pdf file containing a figure showing Kaplan–Meier analysis (MFS) using E2F1 (30th percentile) performed in data subsets with defined adjuvant treatments: (a) none, (b) hormone, (c) chemotherapy and (d) combined. Click here for file
