# Supplementary material for: Low E2F1 transcript levels are a strong determinant of favorable breast cancer outcome
Source: Breast Cancer Res. 2007 May 29;9(3):R33. doi: 10.1186/bcr1681 (PMC1929097; doi:10.1186/bcr1681)
Supplement: Additional File 1 — A Word file containing a table presenting the treatment distribution according to the E2F1 status in both data sets. [file bcr1681-S1.doc]

**Table 1S** Treatment distribution according to E2F1 status in both data sets.

| Adjuvant Therapy | STB Data Set | | NKI Data Set | |
| --- | --- | --- | --- | --- |
|  | **E2F1 p<30** | **E2F1 p>30** | **E2F1 p<30** | **E2F1 p>30** |
| none | 19 (20%) | 41 (19%) | 50 (56%) | 115 (56%) |
| hormone | 50 (53%) | 85 (38%) | 7 (7%) | 13 (6%) |
| chemo | 12 (13%) | 60 (27%) | 27 (30%) | 63 (31%) |
| combination | 11 (11%) | 27 (12%) | 6 (7%) | 14 (7%) |
| total | 73 (77%*) | 172 (77%) | 40 (44%) | 90 (44%) |
| unknown | 3 (3%) | 9 (4%) |  |  |

* % of patients within the respective Data Set and E2F1 subset
