# Supplementary figures and images for: Low E2F1 transcript levels are a strong determinant of favorable breast cancer outcome
Source: Breast Cancer Res. 2007 May 29;9(3):R33. doi: 10.1186/bcr1681 (PMC1929097; doi:10.1186/bcr1681)

# Figure 1S

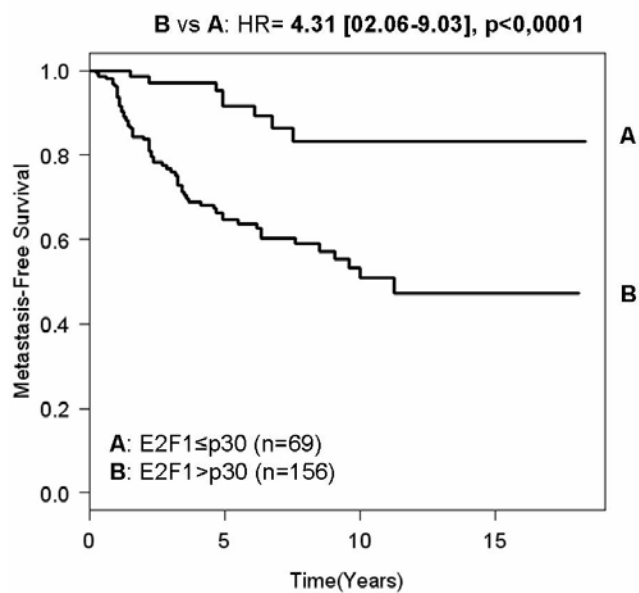

**A**

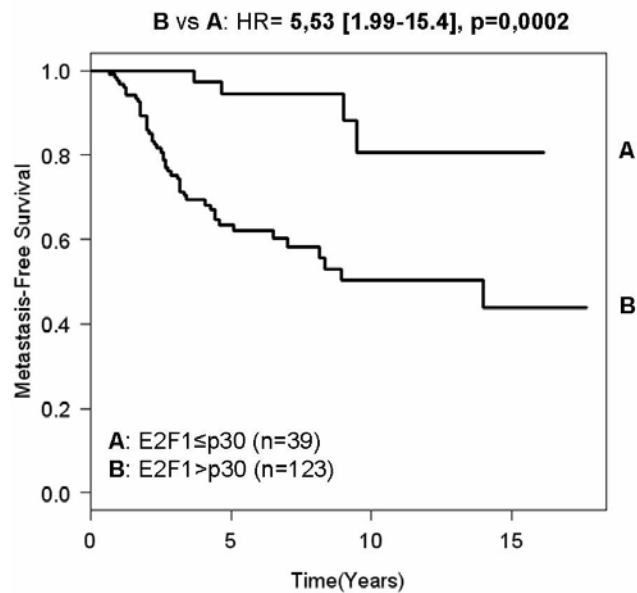

**C**

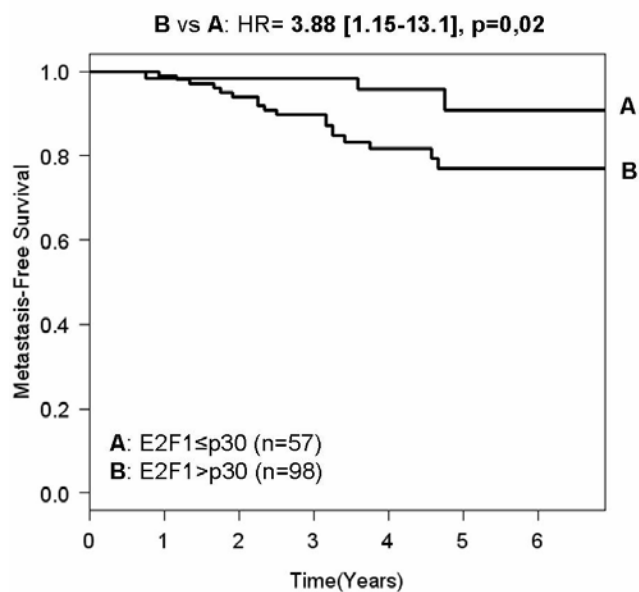

**B**

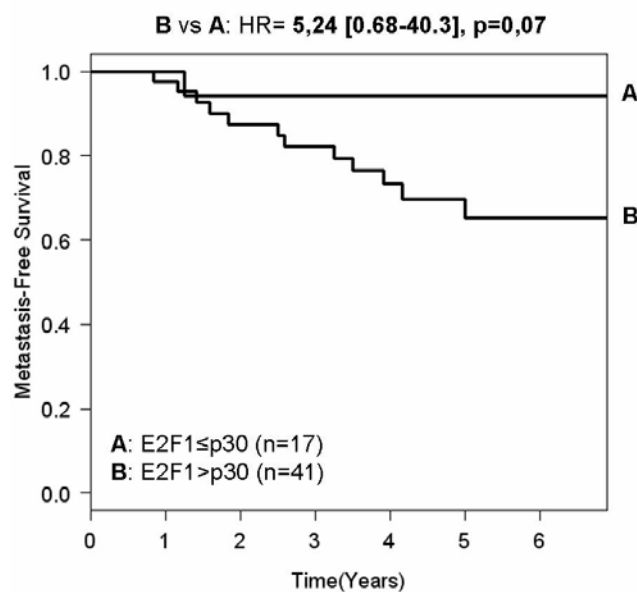

**D**

Supplement: Additional File 2 — A pdf file containing a figure showing Kaplan–Meier analysis (MFS) using E2F1 (30th percentile) performed in data subsets with defined adjuvant treatments: (a) none, (b) hormone, (c) chemotherapy and (d) combined. [file bcr1681-S2.pdf]
